# Psilocybin-assisted therapy in treatment-resistant depression: rapid remission, uncertain durability, and the next phase of clinical evidence

**DOI:** 10.1097/MS9.0000000000005244

**Published:** 2026-06-19

**Authors:** Astrit Sabani, Adnan Khatak

**Affiliations:** a Department of Psychiatry, St. George’s University School of Medicine, St. George’s, Grenada; b Department of Internal Medicine, Nangarhar University, Jalalabad, Afghanistan

**Keywords:** major depressive disorder, neuroplasticity, psilocybin-assisted therapy, psychedelic therapy, sustained remission, treatment-resistant depression

## Abstract

Treatment-resistant depression (TRD) remains a major clinical challenge and is typically defined as the persistence of depressive symptoms despite at least two adequate antidepressant trials. Individuals with TRD experience substantial morbidity, impaired functioning, and elevated suicide risk, highlighting the need for therapeutic strategies beyond incremental refinements of conventional monoaminergic pharmacotherapy. Psilocybin-assisted therapy has recently emerged as a mechanistically distinct intervention, combining transient 5-HT2A receptor agonism with structured psychological support delivered in supervised sessions. Early randomized trials have demonstrated rapid reductions in depressive symptoms and encouraging short-term remission rates, primarily in patients with major depressive disorder, with more limited but emerging evidence in treatment-resistant populations. However, the central clinical question is not merely whether psilocybin can produce acute improvement, but whether these effects can be sustained without cumulative harm. Current evidence remains limited by modest sample sizes, relatively short follow-up periods, challenges in maintaining blinding, and limited comparisons with established TRD interventions such as esketamine, electroconvulsive therapy, and transcranial magnetic stimulation. The durability of benefit beyond several weeks remains an open clinical question, and the implications of repeated dosing are not yet well established. In addition, implementation demands substantial therapeutic infrastructure, raising questions regarding scalability, cost, and equitable access. Future research should prioritize standardized definitions of treatment resistance, recognition of clinical heterogeneity within TRD populations, longer-term outcomes, rigorous active comparators, improved trial methodology, safety monitoring, and cost-effectiveness analyses to determine the appropriate clinical role of psilocybin-assisted therapy.

## Introduction

Major depressive disorder remains one of the leading causes of disability worldwide and contributes substantially to the global burden of disease. A considerable proportion of patients fail to achieve remission despite appropriate pharmacologic management. Treatment-resistant depression (TRD) is commonly defined as the persistence of clinically significant depressive symptoms following at least two adequate trials of antidepressants from different pharmacologic classes, administered at therapeutic doses and durations with confirmed adherence. Individuals meeting criteria for TRD experience disproportionate morbidity, including impaired functional capacity, recurrent hospitalizations, and an elevated risk of suicide. The societal burden is similarly substantial, encompassing both direct health care expenditures and indirect economic losses related to reduced productivity and long-term disability^[^1,2^]^.

Importantly, TRD represents a heterogeneous clinical construct, encompassing variability in illness severity, prior treatment exposure, comorbid conditions, and underlying biological mechanisms. This heterogeneity may influence treatment response and complicate the interpretation and generalizability of emerging clinical trial data.

Over the past several decades, most approved antidepressant medications have targeted monoaminergic neurotransmission, particularly serotonin, norepinephrine, and dopamine pathways. Although incremental improvements in formulation, tolerability, and pharmacokinetics have been achieved, remission rates among patients with treatment-resistant illness remain modest. As a result, successive modifications within the same mechanistic framework have not substantially altered long-term outcomes for a significant subset of patients with refractory depression[2].

In this context, psilocybin-assisted therapy has attracted increasing scientific interest as a mechanistically distinct intervention. Psilocybin, through its active metabolite psilocin, acts primarily as a partial agonist at the serotonin 5-HT_2_A receptor and is administered within a structured therapeutic model that incorporates psychological preparation, supervised dosing sessions, and post-session integration. This approach differs fundamentally from conventional antidepressant strategies based on continuous daily pharmacotherapy. Whether psilocybin-assisted therapy can produce meaningful and durable improvements in individuals with TRD remains an important question requiring careful clinical and methodological evaluation.

## Editorial

### A distinct neurobiological mechanism

Psilocybin differs from conventional antidepressants in both pharmacologic mechanism and therapeutic delivery. Its active metabolite, psilocin, acts primarily as a partial agonist at the serotonin 5-HT_2_A receptor, producing transient alterations in cortical excitability and large-scale brain network dynamics. Unlike selective serotonin reuptake inhibitors, which require sustained daily administration and typically lead to gradual symptom improvement, psilocybin is administered in one or two supervised sessions within a structured psychotherapeutic framework[3].

Preclinical and early human studies have proposed a neuroplasticity-based mechanism of action. Psilocybin exposure has been associated with transient increases in neural flexibility and changes in functional connectivity within large-scale brain networks. Neuroimaging studies suggest modulation of the default mode network, which has been implicated in self-referential processing and maladaptive rumination in depression. Alterations in these network dynamics may temporarily disrupt rigid patterns of neural activity and facilitate cognitive and emotional reappraisal[1].

Clinically, these changes have been conceptualized as an increase in psychological flexibility. However, these mechanistic insights are derived primarily from preclinical and early-phase human studies, and their direct translation to sustained remission remains uncertain. Importantly, the therapeutic model integrates pharmacologic effects with psychological preparation, supervised dosing sessions, and post-session integration. Current evidence does not clearly distinguish the relative contribution of the psychedelic drug effect from that of intensive therapeutic support, and outcomes likely reflect the interaction of both components. Psilocybin-assisted therapy, therefore, represents a time-limited intervention embedded within a broader psychological framework rather than continuous maintenance pharmacotherapy[2].

### Evidence from pivotal clinical trials

The most methodologically rigorous trial evaluating psilocybin in TRD to date is the COMP360 Phase 2b study. In this multicenter trial, adults with TRD were randomized to receive a single 25 mg, 10 mg, or 1 mg dose of psilocybin, administered with psychological support. The primary endpoint was the change in depressive symptom severity at 3 weeks[4].

Participants receiving the 25 mg dose demonstrated significantly greater reductions in depressive symptom scores than those receiving the 1 mg control condition. Approximately 29% of participants in the 25-mg group were in remission at week 3, indicating a rapid antidepressant signal in a treatment-resistant population. However, durability beyond the acute treatment period was more variable, with relapse observed in some participants during the 12-week follow-up. Adverse events were common but generally transient and manageable in supervised settings.

Another randomized study compared two high-dose psilocybin sessions combined with psychological support against daily escitalopram over 6 weeks in patients with major depressive disorder, rather than strictly defined TRD. The trial did not demonstrate the superiority of psilocybin on the primary outcome measure. Several secondary endpoints numerically favored psilocybin, including response and remission rates, although these findings should be interpreted cautiously given the modest sample size and multiplicity concerns[4]. Maintaining effective blinding also remains challenging because of the perceptible psychoactive effects of the intervention.

By comparison, electroconvulsive therapy and transcranial magnetic stimulation have broader evidence bases in TRD, while esketamine has randomized data supporting rapid symptom reduction but often requires maintenance dosing. Psilocybin, therefore, remains at an earlier stage of the evidence-development pathway despite encouraging short-term signals.

Importantly, not all available trials have been conducted in strictly defined treatment-resistant populations, which may limit direct applicability to TRD cohorts. Current studies also evaluate a combined intervention of drug administration and structured psychotherapy, making the independent contribution of each component uncertain. Larger trials with longer follow-up and active comparators are required to determine whether short-term improvements translate into sustained remission in treatment-resistant populations.

### Is rapid remission enough?

TRD is not an episodic condition but a chronic and often relapsing disorder. Many patients experience persistent or recurrent symptoms despite sequential pharmacologic trials, augmentation strategies, and neuromodulation. In this context, rapid symptom reduction is clinically meaningful, particularly for individuals with severe functional impairment or suicidal ideation. However, short-term remission does not necessarily translate into sustained recovery. For an intervention to meaningfully influence treatment paradigms in TRD, durable benefit must remain central to evaluation[2].

Meaningful outcome benchmarks should extend beyond 3 or 6 weeks. Follow-up at 6 months and 12 months better reflects the clinical trajectory of treatment-resistant illness. Experience with ketamine and esketamine illustrates this distinction: rapid antidepressant effects are well documented, yet sustained benefit often requires repeated administration. Similarly, esketamine has established short-term efficacy but is commonly positioned as a maintenance-based treatment rather than a one-time intervention. Whether psilocybin-assisted therapy can produce comparably durable outcomes remains uncertain. Some individuals may require booster sessions, but the optimal frequency and long-term implications of repeated exposure are not yet established[4].

If psilocybin-assisted therapy is to transition from experimental promise to clinical practice, it must demonstrate sustained remission without cumulative psychological or physiological harm. At present, available data are insufficient to determine whether such durability can be consistently achieved in treatment-resistant populations. Ultimately, durability rather than speed alone will determine its long-term clinical role in TRD.

### Safety, patient selection, and ethical considerations

Safety considerations are central to the evaluation of psilocybin-assisted therapy. Clinical trials have generally excluded individuals with bipolar disorder or psychotic spectrum disorders because of concerns regarding mood destabilization and the potential induction of psychotic symptoms. Careful diagnostic screening is, therefore, essential prior to treatment. In controlled clinical settings, observed acute adverse effects have included anxiety, dysphoria, transient perceptual disturbances, and elevations in blood pressure or heart rate. Although these reactions are typically self-limited, the intensity of the experience necessitates continuous monitoring and structured psychological support^[^3,5^]^.

In addition to these observed effects, several potential longer-term risks remain under investigation. Given the elevated baseline risk of suicidality in TRD, systematic assessment of suicidal ideation before and after dosing is particularly important. Psychological destabilization may occur if difficult experiences during the session are not adequately integrated. The therapeutic model is, therefore, resource-intensive and requires trained facilitators capable of managing both the pharmacologic and psychological dimensions of treatment[2].

Ethical considerations also merit attention. Public enthusiasm and media attention surrounding psychedelic therapies may inadvertently inflate patient expectations. Expectancy effects can influence both treatment outcomes and subjective reporting. Individuals with refractory illness may perceive psilocybin as a definitive solution after exhausting conventional therapies. In addition, equitable access may be limited if specialized delivery models remain concentrated in well-resourced centers. Researchers and clinicians must, therefore, communicate the evidence with appropriate caution, emphasizing both the potential benefits and the current limitations of the available data.

### *Implementation and* health*-system realities*

Even if efficacy and safety are confirmed in larger studies, implementation may present substantial practical challenges. Psilocybin-assisted therapy requires trained therapists, structured preparation sessions, prolonged monitored dosing sessions that typically last 6–8 hours, and post-session integration. These requirements distinguish the intervention from conventional pharmacotherapy and substantially increase clinician time commitments^[^1,2^]^.

Economic implications remain uncertain. The combined costs of medication, specialized clinical supervision, and extended therapeutic sessions may limit accessibility. Insurance coverage is also uncertain, particularly in health systems where extended psychotherapy is not routinely reimbursed. Regulatory pathways remain in development, and any approval would likely involve structured risk mitigation frameworks^[^4^]^. Compared with office-based treatments such as conventional pharmacotherapy or standard transcranial magnetic stimulation protocols, the delivery model is comparatively resource-intensive. Empirical data on real-world implementation and cost-effectiveness remain limited.

Consequently, psilocybin-assisted therapy may not be readily scalable in the same manner as conventional antidepressant medications. Its integration into routine care would require deliberate workforce development, reimbursement reform, and strategies to ensure equitable access across health systems.

### Research agenda

If psilocybin-assisted therapy is to be evaluated with the same rigor applied to other interventions for TRD, several priorities should guide the next phase of investigation.

First, standardized staging criteria for TRD are needed. Variability in defining treatment resistance limits comparability across studies and may inflate perceived efficacy in less refractory populations. Clear thresholds regarding the number, adequacy, and sequence of failed interventions should, therefore, be applied consistently.

Second, future studies should incorporate long-term outcome assessments beyond the short horizons typical of current trials. Outcomes at 12 months or longer, with predefined relapse criteria and transparent reporting of maintenance strategies, are more aligned with the chronic and recurrent nature of treatment-resistant illness.

Third, future studies should include active comparators reflecting contemporary standards of care. Head-to-head trials against interventions such as esketamine, electroconvulsive therapy, or transcranial magnetic stimulation would better clarify relative efficacy, durable benefit, and tolerability. Placebo-controlled trials alone are insufficient to determine clinical positioning.

Fourth, methodological innovations in blinding remain important. Because psilocybin produces perceptible psychoactive effects, improved active placebo models or alternative trial designs may help reduce expectancy bias and strengthen internal validity.

Finally, long-term safety monitoring and cost-effectiveness analyses should accompany efficacy studies. Registries tracking repeated exposure and rare adverse events, along with economic evaluations, will be essential for determining whether clinical benefits justify implementation demands. Biomarkers predicting treatment response may also help identify patients most likely to benefit.

## Conclusion

Psilocybin-assisted therapy has demonstrated a credible antidepressant signal in controlled studies, including in selected cohorts of individuals with TRD. Its rapid onset of effect and integration within a structured psychotherapeutic model distinguish it from conventional pharmacotherapy. At the same time, important uncertainties remain regarding the durability of benefit, comparative effectiveness, long-term safety, and real-world scalability. The field, therefore, stands at a critical stage of development. Carefully designed trials with longer follow-up, rigorous comparators, and transparent safety monitoring will determine whether psilocybin-assisted therapy ultimately becomes a specialized option for selected patients or contributes to a broader shift in the management of refractory depression. At present, its precise clinical positioning remains to be established. Balanced appraisal, rather than enthusiasm alone, will be essential in defining its appropriate clinical role.

## Data Availability

No new datasets were generated or analyzed in the preparation of this editorial. All information discussed in the manuscript is derived from previously published studies cited in the reference list.
